# Therapeutic potential of *Coprinus comatus* nanogels: Antiarthritic and anti-inflammatory effects in rheumatoid arthritis models

**DOI:** 10.14202/vetworld.2025.582-597

**Published:** 2025-03-09

**Authors:** Nuniek Ina Ratnaningtyas, Fajar Husen

**Affiliations:** 1 Department of Biology, Faculty of Biology, Universitas Jenderal Soedirman (UNSOED), Purwokerto 53122, Central Java, Indonesia; 2 Department of Medical Laboratory of Technology, Bina Cipta Husada College of Health Science, Purwokerto 53144, Central Java, Indonesia

**Keywords:** anti-inflammatory, *Coprinus comatus*, cytokine reduction, herbal medicine, nanogels, rheumatoid arthritis

## Abstract

**Background and Aim:**

Rheumatoid arthritis (RA) is a chronic autoimmune disorder characterized by persistent joint inflammation and systemic immune dysregulation. The current pharmacological treatments, primarily synthetic drugs, often present adverse effects and long-term toxicity. This study explores the therapeutic potential of *Coprinus comatus* nanogels as a novel herbal formulation with antiarthritic and anti-inflammatory properties in a Complete Freund’s Adjuvant (CFA)-induced rat model of RA. The study aims to evaluate the efficacy of *C. comatus* nanogels in reducing pro-inflammatory cytokine levels, antibody production, paw edema, and arthritis indices and to assess their potential as a safer alternative to conventional RA therapies.

**Materials and Methods:**

Twenty-four male Wistar rats were randomized into six groups: Healthy control, negative control (CFA-induced without treatment), positive control (sodium diclofenac 0.012 g/mL), and three treatment groups (TG1, TG2, and TG3) receiving 250, 500, and 750 mg/kg *C. comatus* nanogels, respectively. Oral treatments were administered for 30 days. Pro-inflammatory cytokines (tumor necrosis factor-alpha [TNF-α], interleukin [IL]-6, IL-1β), antibodies (immunoglobulin [Ig]G, IgE), cyclooxygenase-2 (COX-2) enzyme activity, paw edema, and arthritis indices were measured using enzyme-linked immunosorbent assay and standard methods. Statistical analyses were conducted using one-way analysis of variance.

**Results:**

The 750 mg/kg dose of *C. comatus* nanogels significantly reduced TNF-α (17.71%), IL-1β (19.83%), and IgE (23.91%) levels. The 250 mg/kg dose exhibited the highest reductions in IL-6 (30.88%) and COX-2 (16.54%) levels. TGs demonstrated a 27.75% reduction in paw edema and a 45.45% reduction in arthritis indices. Key bioactive compounds contributing to these effects included flavonoids, polyphenols, triterpenoids, and β-glucans.

**Conclusions:**

*C. comatus* nanogels demonstrated promising anti-inflammatory and antiarthritic properties, suggesting their potential as an alternative herbal treatment for RA. Further studies are recommended to explore the long-term safety and clinical applicability of *C. comatus* nanogels in human RA management.

## INTRODUCTION

Inflammation is a biological process mediated by the entry of antigens, such as pathogens and toxic chemical compounds. Inflammation can impair health in the form of degenerative or immune diseases [1]. One autoimmune disease also affected by inflammation is rheumatoid arthritis (RA). RA is a chronic disease with a global prevalence of nearly 1%, with the highest prevalence observed in female patients. Until now, the leading cause of RA has not been explained with certainty and detail, but what is associated with RA is bone and cartilage damage caused by autoimmune and inflammatory mechanisms. A characteristic observed in patients with RA is an increase in pro-inflammatory cytokine levels [2]. Some experts also mention that the cause of RA is related to environmental and genetic factors. Previous research by Joo *et al*. [3] has reported that environmental factors such as cigarette smoke and exposure to heavy metals such as cadmium and lead can increase the risk of developing RA disease. The pathophysiological mechanism in patients with RA begins when joint deformity occurs, which causes several effects, such as bone erosion, atrophy of the skeletal muscles, invasion of synovitis, and damage to tendons and ligaments [4]. Increasing the levels of pro-inflammatory cytokines in people with RA can increase the risk of inflammation that causes permanent joint damage. The initial stage of inflammation is characterized by swelling around the joints and synovial membrane. This will cause severe pain and interfere with joint movement. The proinflammatory cytokines found in RA inflammation are interleukin-6 (IL-6) and tumor necrosis factor-alpha (TNF-α). In addition, cytokines such as IL-17, IL-1β, IL-1, and interferon-gamma (IFN-γ) are released, leading to chronic inflammation [5]. Other inflammatory mediators released during inflammation in patients with RA can cause the proliferation of fibroblast-like synoviocytes (FLS) and abnormal proliferation [6]. Research to reveal the pathophysiology of RA disease has begun using experimental animals. This study focused on the induction of Complete Freund’s Adjuvant (CFA) in an animal model. The use of CFA is performed because it can lead to chronic inflammatory effects that can cause an increase in pro-inflammatory cytokines as well as significant physiological changes and systemic effects on experimental animals in the form of hyperplasia due to the release of cytokines [1]. Hyperplasia after CFA induction is due to changes in transduction sensitivity in the induced tissue. In addition to hyperplasia, severe pain such as burning, stinging, or allodynia can also occur [7]. Observations of pro-inflammatory cytokines are insufficient to describe the inflammatory process occurring under RA conditions; therefore, antibody measurements and other inflammatory mediators, such as cyclooxygenase (COX), are performed. The observation of COX is important because COX, especially COX-2, plays a role in the inflammatory process that releases prostaglandins (PG), especially prostaglandin E2 (PGE2) [8]. PGE2 plays a significant role in edema formation; therefore, this study also measured edema volume to correlate the effect of increased COX-2 levels with edema.

RA treatment requires a long time and high consistency because it is an auto-immune disease with many causative factors and complicated pathophysiological processes and is characterized by many immunological signaling pathways [4]. The use of natural resources has begun to be widely developed to identify biological, chemical, or natural substances with potential anti-inflammatory and anti-arthritis properties. Most of the exploration and testing of herbal products with potential anti-inflammatory and anti-arthritis properties have focused on condensed extracts [9]. While the development of preparations in nanogels is still rare, disclosing the potential of natural resources in the form of fungi is still not as common as testing using herbal plants. Thus, this study focuses on testing nanogels from *Coprinus comatus* mushrooms as anti-arthritis and anti-inflammatory agents in CFA-induced experimental animals. *C. comatus* has been recognized as a potential mushroom for herbal medicine because of its bioactive compounds, such as quercetin, rutin, ascorbic acid, α-tocopherol, polyphenols, flavonoids, and alkaloids [10]. One of the bioactive compounds of *C. comatus* that can act as anti-inflammatory agents is quercetin and polyphenols. These compounds can suppress free radicals formed during inflammatory reactions to prevent cell membrane lipid peroxidation due to oxidative stress and increased proinflammatory cytokine levels [11]. Ratnaningtyas *et al*. [1] showed that applying nanogels from *Ganoderma lucidum* mushrooms reduced IL-6, TNF-α, and IL-1β levels using nanogel doses of 500 and 750 mg/kg body weight (BW). The use of nanogels as a dosage form is given orally in *in vivo* experiments because this application is still very rare. In addition, applying *G. lucidum* nanogels can reduce COX-2 levels, an enzyme that plays a role in the pathophysiology of RA [1]. The use of nanogel preparations maintains bioactive compound composition in *C. comatus*. Preparations in the form of extracts can affect the stability of bioactive compounds if the storage conditions are not appropriate in the long term. Hence, the application of *C. comatus* nanogels should be further developed. The application of nanogel preparations is also more flexible and can respond very well to changes in pH and temperature compared to preparations in the form of viscous extracts [12]. In addition, the use of the fruiting body of the *C. comatus* mushroom as the main ingredient is advantageous because making preparations from the fruiting body is easier than mycelial culture. The fruiting body of *C. comatus* contains many polysaccharide compounds such as (Ccp-I-A, Ccp-I-B) which can prevent lipid peroxidation reactions due to inflammation and the breakdown of polyunsaturated fatty acid chains of cell membranes due to the high activity of these polysaccharides as antioxidants and anti-inflammatories [13]. The long-term use of synthetic drugs that can cause side effects, increase toxicity, and decrease immunity (immunosuppressant) is something that must be considered, so it is necessary to develop alternative herbal medicines that are safer and have minimal side effects [14]. Research using *C. comatus* mushrooms as antiarthritic and anti-inflammatory is still not widely done. The application of *C. comatus* mushrooms is still limited to testing as antidiabetics, antioxidants, antibacterials, and anticancer agents, so further investigation is needed regarding the potential of *C. comatus* bioactive compounds as antiarthritis and anti-inflammatory agents *in vivo*. The novelty of this study is that in addition to using preparations in the form of nano gels, an evaluation was also carried out regarding the antiarthritic effect of *C. comatus* in reducing COX-2 levels, rat paw edema, and arthritis index (AI). Meanwhile, anti-inflammatory evaluation was performed by measuring pro-inflammatory cytokine levels (TNF-α, IL-6, and IL-1β) and antibody levels in serum (immunoglobulin [Ig]G and IgE). The *C. comatus* mushroom used in this study was locally cultivated in Indonesia, especially in the Cianjur area, where the source of isolates comes from forest areas in Cianjur Regency, West Java. This *C. comatus* mushroom is slightly different from those found in China or Japan, where the size is relatively smaller and the stamp size is more rounded. The growing medium used in *C. comatus* is also different, with the addition of corn husks and bran.

The development of nanogel formulations can also provide advantages in research because nanogel preparations are easily soluble, so they are easy to administer orally and allow faster absorption. Extract formulations often leave solvent residues, and extract preparations that are still thick are also not easily dissolved. In addition, the storage of extracts must be considered because they are easily contaminated with fungi. The storage of extracts must be considered so that the bioactive compounds are not damaged or oxidized because extract preparations are usually moist [15]. Nanogel preparations have better biochemical stability than extracts, and because of their nanosize, herbal or medicinal preparations in the form of nanogels will more easily reach the target tissue; thus, nanogel preparations have good potential in therapeutic testing applications [16]. Other studies also mentioned that nanogel applications have better effectiveness because of their high surface area and free energy. The toxicity of nanogels is considered to be lower, and due to the incorporation of hydrophilic compounds, the absorption process of the preparation will be better [17]. Research related to the use of *C. comatus* mushrooms as anti-inflammatory agents has not been widely conducted. Previous research only revealed the potential of *C. comatus* as an anti-diabetic and antioxidant supplement using various types of extracts, such as ethanol, water, and ethyl-acetate extracts. However, in this research, we used another formulation using nanogels with the advantage of easier nanogel storage, more complex formulations, and not easily contaminated [1]. Research related to the potential of *C. comatus* as an antiarthritis and anti-inflammatory agent is still very rare, so it is necessary to reveal the relationship between RA and inflammation, considering that the pathogenesis of RA is strongly influenced by inflammatory reactions. We developed a nanogel formulation based on our previous research using the ethanol extract of *C. comatus* as an anti-inflammatory and immunosuppressant in carrageenan-induced inflammatory model rats. Based on the results of previous research by Ratnaningtyas *et al*. [11], the ethanol extract of *C. comatus* can reduce the levels of IL-1β, IgE, nitric oxide (NO^-^), edema, plantar thickness of animal feet, and the number of leukocytes. However, the parameters in this study are still limited to the application of extracts and incomplete cytokine parameters. Thus, we conducted further research with a focus on the application of nanogels and the determination of more complete inflammatory parameters.

Therefore, this study aimed to examine and investigate the therapeutic potential of *C. comatus* mushrooms in nanogel preparations as anti-arthritis and anti-inflammatory agents by identifying the content of biological compounds in *C. comatus* nanogels, measuring the levels of pro-inflammatory mediators, antibodies, and enzymes that play a role in inflammation, and measuring the inflammatory effects in *in vivo* experiments in the form of index arthritis and edema. It is hoped that this research will contribute to the development of alternative herbal medicines that are safe, natural, and can be used in the long term to suppress inflammation.

## MATERIALS AND METHODS

### Ethical approval

The treatment of experimental animals has received ethical approval from the Health Research Ethics Commission (KEPK) of the Faculty of Medicine, Universitas Jenderal Soedirman, with approval certificate 030/KEPK/PE/II/2023. The inclusion and exclusion criteria of experimental animals were adjusted to the ethics commission rules, and the acclimatization, experimentation, and termination of experimental animals were carried out according to the applicable standard operating procedures (SOPs). The treatment process also refers to the ethical principles of replacement, reduction, and refinement (3 R); the freedom from hunger and thirst, freedom from discomfort, freedom from pain, injury, and disease; the freedom from fear and distress; and the freedom to express natural behavior (5 F) by the rules of an Institutional Animal Care and Use Committee. In this study, the 3R principles of replacement, reduction, and refinement were considered. The replacement principle was carried out by avoiding the use of excessive experimental animals as much as possible (such as replacement due to death), as well as by paying attention to *in vivo* testing of mice compared to directly testing larger mammals/humans. The reduction principle was carried out by paying attention to the needs as much as possible to obtain valid data without using a large number of experimental animals. This principle was also carried out by taking into account the minimum needs, including the estimated dropout according to the calculation of the feeder formula. Refinement is a principle that is carried out by considering maintenance management, feeding, and modification of housing so that the experimental animals do not experience stress, fear, and discomfort.

### Study period and location

The study was conducted from March to November 2023 at the Research Laboratory of the Faculty of Medicine and Faculty of Biology, Universitas Jenderal Soedirman (UNSOED), Purwokerto, Central Java, Indonesia.

### Materials

The mushroom used was a *C. comatus* (O.F. Müll.)Pers. Nuniek Ina Ratnaningtyas (Professor of Applied Mycology, Faculty of Biology, Jenderal Soedirman University) and Triyono Untung Priyadi (Scientist CV. Asa Agro Corporation, Cianjur, West Java) identified the mashrooms with Herbarium No. Coprinus/001/MedMushroom/2018. Fruiting body of *C. comatus* cultivated and harvested from CV Asa Agro Corporation (AAC), Cianjur Regency, West Java, Indonesia. Twenty-four male Wistar rats (*Rattus norvegicus*) weighing 200 g and aged 3–4 months were purchased from UD. Wistar, Special Region of Yogyakarta, Indonesia. Enzyme-linked immunosorbent assay (ELISA) kits for the main parameter analysis were obtained from Shanghai Korain Biotech Co. Ltd., China, including TNF-α (catalog no. E0764Ra), IL-6 (catalog no. E0135Ra), IL-1β (catalog no. E0119Ra), COX-2 (catalog no. E1680Ra), Rat. ELISA kit IgG (catalog no. EA0033Ra), IgE (catalog no. E0452Ra), feed pellets for rats (RatBio Citra Feed PT. Citra Feedmill, East Jakarta, Indonesia), and raw rice husks. The chemicals used were 0.9% physiological NaCl (500 mL @PT. Widatra Bhakti, Pasurua, East Java, Indonesia), alcohol 95% (@One Med 1000 mL), and 0.012 g/200 g BW sodium diclofenac (Bernofarm Pharmaceutical PT. Sidoarjo, East Java, Indonesia), furthermore, 2500 mL of absolute ethanol solvent (pro-analysis 100% concentration), 0.012 g of CFA (Sigma Aldrich Product No. F5881-10ML, Massachusetts, United States), and other materials, including ether and distilled water. The tools used were a 50 × 30 × 20 cm rearing cage (MKV-1127200205MDL @PT. Mitra Kultiva Utama, Bogor City, Indonesia), vacuum rotary evaporator (RE-2010 Henan Lanphan Industry Co. Ltd.Zhengzhou, China), ELISA reader (FlexA-200@ThermoFisher,Jakarta, Indonesia), microscope (Olympus CX-23,Tokyo, Japan), and hematocrit capillary (Nesco Microhematocrit Capillary (non-heparinized) 1.1–1.2 mm,Jakarta, Indonesia). Gas-chromatography mass spectrometry (GC-MS) (Q Exactive™ GC Orbitrap™ Gas Chromatography Tandem Mass Spectrometry, ThermoFisher, Jakarta, Indonesia), and water bath (Precision™ Circulating Water Bath catalog no.TSCIR35, Jakarta, Indonesia).

### Mushrooms extraction

Mushroom extraction was performed using the maceration method. The solvent used was absolute ethanol. A total of 500 g of dry simplicia powder were weighed and mixed with 1500 mL of ethanol solvent. On the next day, remaceration was carried out using the ratio of simplicia to solvent, namely 1:1 and 3:1. The total macerate was then filtered and evaporated using a rotary vacuum evaporator to obtain a thick extract. The temperature used in the evaporation process was 78°C. The thick extract obtained was then stored at a temperature of 5°C [18].

### Nanogel formulations

Nanogels were prepared by mixing the viscous extract of *C. comatus* for each dose to be made (250, 500, and 750 mg/kg BW). *C. comatus* nanogels were prepared using a sonification technique with 40–50 kHz ultrasonic waves. The nanoemulsions were prepared using the gelling agent carbopol 940. The oil phase was prepared by gradually adding isopropyl myristate to the surfactant solution (Tween 80). Next, methyl paraben and propyl paraben solutions were added until homogeneous. The surfactant was added in the form of propylene glycol solution and distilled water until homogeneous. The process was carried out in a beaker glass with homogenization using a magnetic stirrer at 150× *g* for 15–30 min. Incubation was performed for 24 h until a clear nanoemulsion solution was obtained. The next stage of gel-base preparation involved developing carbopol 940 with distilled water. The development process took 24 h. The carbopol concentration ranged from 0.5% to 2.5% [17].

The nanoemulgel formulation was prepared by mixing the gel base and nanoemulsion preparation formula in a beaker glass, homogenized for 30 min at 150× *g*, and stirred using a magnetic stirrer. The composition used included thick *C. comatus* mushroom extract, isopropyl myristate, tween 80, propylene glycol, methyl paraben, propyl paraben, and distilled water. The gel ingredients were carbopol 940, triethanolamine, and distilled water. The preparation was evaluated by testing organoleptic parameters (nanogel appearance, color, odor, and shape), homogeneity level, acidity value (pH) in the range of 5–7, viscosity level, nanogel spreadability level, and freeze and thaw testing value. Dosage determination was performed based on previous research by Ratnaningtyas *et al*. [1] and dose conversion, in which doses of 250–750 mg/kg BW reduced the levels of pro-inflammatory cytokines (TNF-α).

### Mycochemicals of *C. comatus*

Qualitative and quantitative identification of the mycochemical compounds in the thick extract was performed. The target compounds were flavonoids, alkaloids, triterpenoids, and saponins. Flavonoid identification was performed using an amyl alcohol reagent with the addition of magnesium powder (Mg^2+^) as well as hydrochloric acid (HCl) solution (a color change to yellow, reddish yellow marks positive sign). Alkaloid identification was carried out using Mayer-Dragendorff-Bouchardat reagent (marked by a change in color to pink, dark pink), and triterpenoid identification was carried out using concentrated sulfuric acid (H_2_SO_4_) and acetic acid anhydrite (CH_3_COOH) (a positive sign is a change in color to dark purple, purple-black). The identification of saponins was performed using boiling with the addition of HCl, and a positive sign characterized by the formation of a stable foam was obtained for 30 s [9].

Quantitative identification was performed using Agilent 6980 N GC-MS. The ethanol extract of *C. comatus* was dissolved in ethanol. The detector used was Agilent 5973 inert Mass Selective Detector. *C. comatus* extract samples that have been dissolved are injected as much as 1.5–2.0 μL into the injection column (J&W Scientific HP-5MS); the mobile phase used was acetonitrile. The reading results were then computerized and compared with scientific data in the PubChem National Center for Biotechnology Information (NCBI) database (https://pubchem.ncbi.nlm.nih.gov/), ChemSpider database (https://chemspider.com), and Spectrabase (https://spectrabase.com). The results of quantitative GC-MS measurements included alkaloids and flavonoid compounds [11].

### Induction of CFA

The CFA solution dissolved 2 g of CFA powder in 10 mL of distilled water. CFA induction was performed intraplantar with a volume of 0.1 mL on the soles of the feet of the experimental rats. The soles of the rat feet were cleaned using 80% alcohol before induction. The rat’s foot was held and placed on a cushion so that it did not move freely. The injection was then performed slowly. After induction, edema and arthritis were observed in rats for 30 days [1].

### Animals and nanogel treatment

The post-test method was used, and the control group was designed with a complete randomized design. The number of experimental animals was 24, consisting of male Wistar rats (12 weeks old with a BW of 200 g). The use of male rats in *in vivo* tests is because they are hormonally more stable and are not influenced by the estrous cycle as in female rats. Therefore, the measurement of anti-arthritis and anti-inflammatory parameters is expected to be more valid. The rats were divided into six groups: Healthy control (HCt, without treatment), negative control (NCt, induced CFA 0.1 mL), positive control (PCt, induced CFA 0.1 mL, and given sodium diclofenac 0.012 g/kg BW), and treatment group 1 (TG1, induced CFA 0. 1 mL, and given *C. comatus* nanogel 250 mg/kg BW), treatment group 2 (TG2, induced CFA 0.1 mL, and given *C. comatus* nanogel 500 mg/kg BW), and treatment group 3 (TG3, induced CFA 0.1 mL, and given *C. comatus* nanogel 750 mg/kg BW). Nanogel and diclofenac sodium were administered after CFA induction and were administered for 30 days, once every morning after meals (at 09:00) [1]. The use of diclofenac sodium as a PCt was chosen because this drug is very commonly used for treating RA to treat pain and inflammation. Diclofenac sodium is also sold freely in pharmacies, so it is readily available. In addition, diclofenac sodium is classified as non-steroidal anti-inflammatory drug that inhibit the synthesis of COX-2 and PGE2 and reduce the risk of RA inflammation [19]. Therefore, the use of diclofenac sodium is related to the parameters measured in this study, namely COX-2 levels. The dose selection was based on previous research using *C. comatus* extract in *in vivo* experiments with carrageenan induction and on dose conversion according to the BW of the experimental animals. In the use of *C. comatus* extract, doses of 500 and 750 mg/kg BW gave a good reducing effect on IL-1β and IgG levels [11], so in this research with various anti-inflammatory response, parameters such as COX-2, IgE, IL-6, and TNF-α were maintained with the hope that nanogel doses similar to extracts could give good effects. With an increase in the three doses used, it is hoped that the optimal dose will be found as anti-arthritis and anti-inflammatory.

### Animal treatment

The experimental animals were acclimatized for 7 days under controlled environmental conditions (temperature 27–30°C, humidity 65%–70%, and lighting frequency 8–10 h, cleaning the drum every 3 days, and disinfecting the drum before use using 80% alcohol disinfectant). Groups of rats were then weighed to meet the inclusion and exclusion criteria. The BW of the rats used was 200–210 g. Feeding was performed twice a day, every morning (at 08.00) and afternoon (at 16.00), and drinking water was changed every 3 days *ad libitum* [20].

### Sample collection

Blood sampling was performed on the 30^th^ day after the administration of the *C. comatus* nanogel was completed. Before the blood was taken, the experimental animals were fed for 5–8 h; blood was taken using a hematocrit capillary pipette, then inserted in an ethylenediaminetetraacetic acid vascular tube (OneMed K3 3mL PET, Jayamas Medica Industry PT., East Java, Indonesia), and centrifuged (Multi-Purpose DM 0636 DLAB – 150× *g*) for 5–10 min. The serum was then collected and stored at 5°C using a microcentrifuge tube (Indo Lab Utama PT. Catalog no. MCTB020-R, Jakarta, Indonesia). The experimental animals were terminated by decapitation of the neck and anesthesia using a combination solution of ketamine and xylazine. Furthermore, the animals were buried in a place that had been provided and away from settlements.

### Parameters analysis

The ELISA method was used to measure and analyze key parameters, including TNF-α, IL-1β, IL-6, COX-2, IgE, and IgG. ELISA analysis procedures were performed according to the manufacturer’s SOPs. Samples were analyzed with an ELISA Reader at a wavelength of 410 ± 10 nm (TNF-α, IL-1β, IL-6, COX-2, IgE, and IgG). The parameters of paw edema volume and AI were measured using a plethysmometer over 30 days [1].

### Statistical analysis

All experimental data were analyzed using the statistical software Statistical Package for the Social Sciences (version 26.0, IBM Corp., NY, USA) and GraphPad Prism (version 10.2.3, GraphPad Software, Inc., San Diego, CA, USA). Results are expressed as mean ± standard error of four replicates per group. Statistical significance was determined using one-way analysis of variance to assess differences among the experimental groups. Where significant differences were detected (p < 0.05), *post hoc* multiple comparisons were performed using Duncan’s multiple range test to identify specific group differences.

The parameters analyzed included pro-inflammatory cytokines (TNF-α, IL-6, IL-1β), antibodies (IgG and IgE), and inflammatory markers, such as COX-2, paw edema volume, and AI. The percentage reduction for each parameter was calculated relative to the NCt group to highlight the therapeutic effects of the *C. comatus* nanogels. Graphical representations of data were created using GraphPad Prism (version 10.2.3) to visualize trends and significant differences between groups.

This statistical approach ensures a rigorous evaluation of the therapeutic efficacy of *C. comatus* nanogels in modulating inflammation and arthritis-related markers, providing a robust framework for interpreting the study findings [21].

## RESULTS

### Qualitative and quantitative identification results

Based on the research results, at the extraction stage of *C. comatus* using ethanol, 1000 g of dry powder simplicia of *C. comatus* produced as much as 18 g of thick extract. The qualitative analysis results of the mycochemical compounds of *C. comatus* are presented in Table 1.

**Table 1:** Qualitative identification of the cytochemical compounds of *Coprinus comatus*.

Mycochemicals	Reagent/tester compound	Result	Quantitative results (mg/g)
Triterpenoids	Acetic acid (CH_3_COOH) + Sulfuric acid (H_2_SO_4_)	+ (Purple)	1.47
Polyphenols+Tannins	FeCl_3_ 10%	++ (Blue)	—
Flavonoids	Zn/Mg powder+Amyl alcohol+HCl	++ (Reddish orange)	1.51
Alkaloids	Mayer, Dragendorff, Bouchardat (dye)	++ (Dark brown)	1.52
Minerals (Phosphoric acid)	—	—	10.21
Fatty acid	—	—	3.13

The dashed line indicates that the data were not qualitatively/quantitatively analyzed. A positive sign indicates the presence of the target compound in the extract: + (moderate level), ++ (considerable level), or +++ (abundant level)

Table 1 shows that the ethanol extract of *C. comatus* contains triterpenoid compounds, polyphenols, flavonoids, alkaloids, minerals, and fatty acids. The highest result based on quantitative analysis with GC-MS was for mineral compounds with more than 10.21 mg/g extract, while the least amount is triterpenoid with 1.47 mg/g extract. The qualitative analysis also showed similar results, where one positive sign was obtained for identifying and screening triterpenoid compounds. Based on the GC-MS results compared with the compound database on PubChem NCBI (https://pubchem.ncbi.nlm.nih.gov/), ChemSpider database (https://chemspider.com), and Spectrabase (https://spectrabase.com), 10 compounds were identified, as presented in Tables 2 and 3.

**Table 2:** GC-MS identification and analysis results.

No.	Compound name	Molecule formula	Retention time	Molecule weight (g/mol)	% Area
1	Phosphoric acid, diethyl 2-(ethylthio) ethyl ester (CAS)	C_8_H_19_O_4_PS	20.98	242.47	1.04
2	Tetralin-1-methylamine, N-cyclohexyl-N-oxide-	C_17_H_23_NO	24.475	257.38	0.07
3	Butyronitrile, 4,4,4-trifluoro-2-methyl (CAS)	C_5_H_4_F_3_NO	23.741	151.08	0.08
4	Thiophene-3-carbonitrile, 2-methylthio-5-(1-piperidinylcarbonyl)-4-(2-thienyl)-	C_5_H_3_NS	22.655	109.13	1.03
5	Ketone, methyl 2,4,4,5,5-pentamethyl-1-cyclopenten-1-yl, semicarbazone (CAS)	C_13_H_23_N_3_O	22.70	237.34	2.31
6	1-(4-amino-1,2,5-oxadiazol-3-yl)-5-methyl-1h-1,2,3-triazole-4-carboxylic acid	C_8_H_11_N_7_O_3_	23.858	253.22	0.10
7	5,9-Methanobenzocyclooctene, 3-chloro-5,6,9,10-tetrahydro-5,7,8,9-tetramethyl-	C_17_H_21_Cl	23.905	260.81	0.22
8	2-Octenoic acid, 7-hydroxy-, ethyl ester (CAS)	C_10_H_18_O_3_	22.775	186.25	1.34
9	Phosphorothioic acid, O, O-diethyl O-[2-(1-hydroxy-1-methylethyl)-6-methyl-4-pyrimidinyl] ester (CAS)	C_12_H_21_N_2_O_4_PS	21.66	320.35	1.44
10	Lupan-3-one (CAS)	C_30_H_50_O	24.06	426.21	0.08

GC-MS=Gas chromatography–mass spectrometry, CAS=Consolidated account statement

**Table 3:** Chemical structure of mycochemical compounds.

Compounds name	Chemical structure	Compounds name	Chemical structure
Diazinon	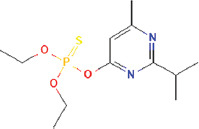	Thiophene-3-carbonitrile	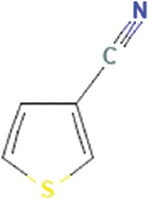
Tetralin methylamine	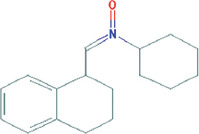	Ketone semicarbazone	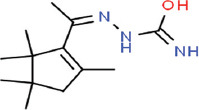
Butanenitrile trifluoromethyl	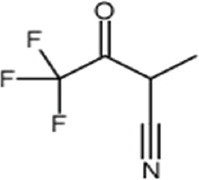	Carboxylic acid	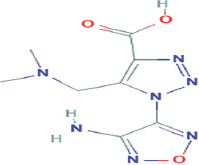
Methanobenzocycloocten	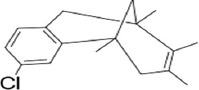	Octenoic acid	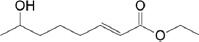
Phosphorothioic acid	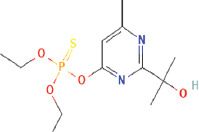	Lupanone	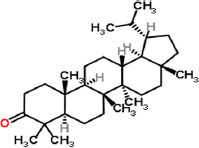

**Figure 1:**
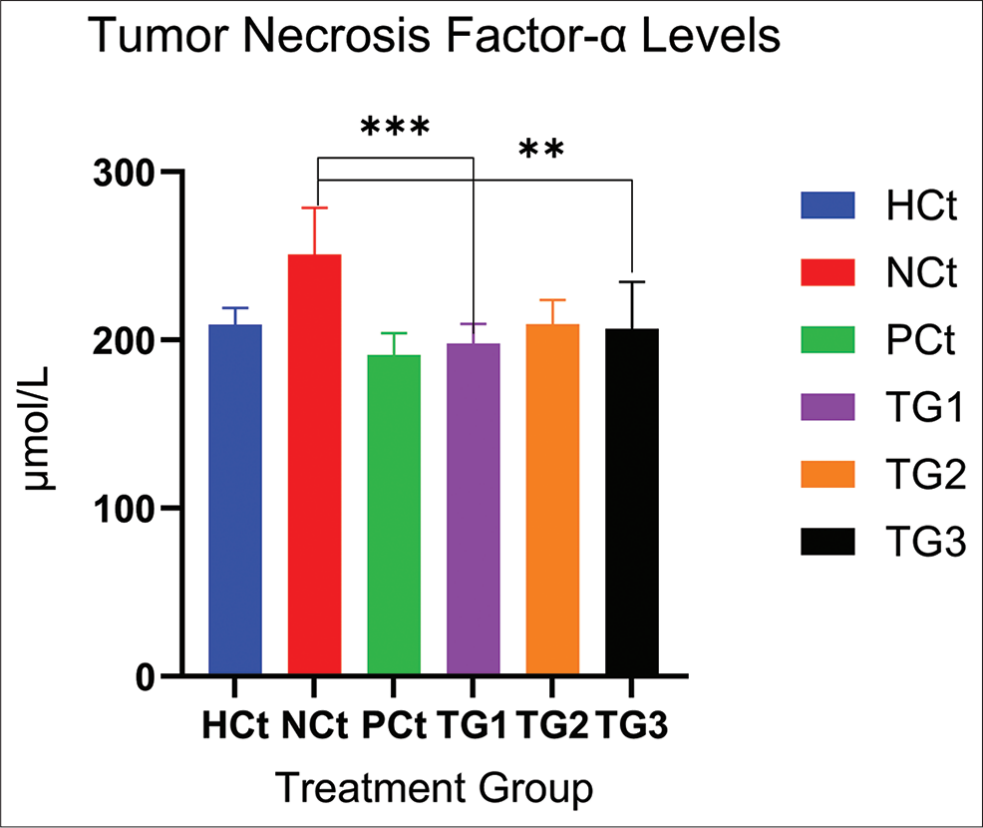
TNF-α levels after treatment. A significant increase (p < 0.05; *: indicates significance). Data are expressed as mean ± SD (n: 4). HCt=Healthy control, NCt=Negative control (CFA-induction only), PCt=Positive control (administration of sodium diclofenac 0.012 g/mL), TG1=Administration of 250 mg of *C. comatus* nanogel, TG2=Administration of 500 mg of *C. comatus* nanogel, TG3=Administration of 750 mg of *C. comatus* nanogel for 30 days, TG=Treatment groups, TNF-α=Tumor necrosis factor-alpha, CFA=Complete Freund’s Adjuvant, SD=Standard deviation, *C. comatus*=*Coprinus comatus*.

Tables 2 and 3 present the cytochemical information of the *C. comatus* ethanol extract based on GC-MS results. The highest retention value was for the compound Tetralin-1-methylamine, N-cyclohexyl-N-oxide, while the highest molecular weight was for phosphorothioic acid. The lowest retention time was for the phosphoric acid diethyl 2-(ethylthio)ethyl ester, while the most negligible molecular weight was for the thiophene-3-carbonitrile. Many mycochemical compounds identified by GC-MS are classified as inorganic compounds, organic compounds, minerals, and fatty acids (unsaturated). Examples of compounds that include fatty acids are phosphorothioic acid and octenoic acid, whereas compounds that include mineral acids are phosphoric acid. Some mycochemical compounds of *C. comatus* ethanol extract contain hydroxyl groups, such as ketone semicarbazone and octanoic acid. Proinflammatory cytokine measurement results (TNF-α, IL-6, and IL-1β). The results of measuring TNF-α levels showed that the administration of *C. comatus* nanogels for 30 days had a significant decreasing effect (p < 0.05). TNF-α levels after nanogel administration in other experimental groups are presented in Figure 1.

**Figure 2:**
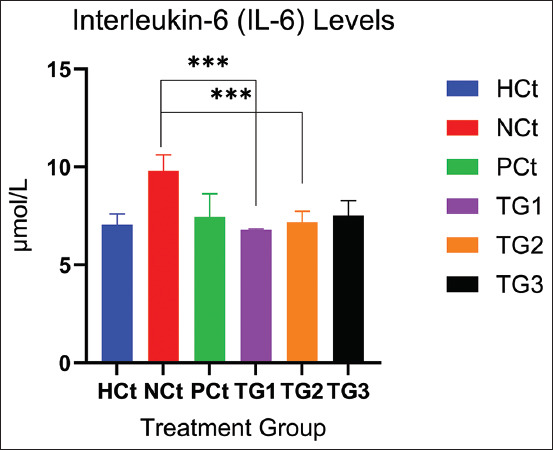
IL-6 levels after treatment. A significant increase (p < 0.05; *: indicates significance). Data are expressed as mean ± SD (n: 4). HCt=Healthy control, NCt=Negative control (CFA-induction only), PCt=Positive control (administration of sodium diclofenac 0.012 g/mL), TG1=Administration of 250 mg of *C. comatus* nanogel, TG2=Administration of 500 mg of *C. comatus* nanogel, TG3=Administration of 750 mg of *C. comatus* nanogel for 30 days. TG=Treatment groups, IL=Interleukin, CFA=Complete Freund’s Adjuvant, SD=Standard deviation, *C. comatus*=*Coprinus comatus*.

The decrease in TNF-α levels compared with the NCt group with the TG significantly occurred in the TG1 and TG3 groups. The highest decrease was observed in the TG1 group (21.18% (197.83), TG2 16.54% (209.48 μmol/mL), and TG3 17.72% (206.51 μmol/mL). TNF-α levels in the NCt group were >250 μmol/mL, whereas diclofenac sodium administration showed a decrease of 23.85%. In addition, a decrease in proinflammatory cytokine levels was also observed in the results of IL-6 measurements presented in Figure 2.

The IL-6 levels in all experimental groups were significantly decreased (p < 0.05). Administration of *C. comatus* nanogels for 30 days reduced IL-6 levels by 30.89% (6.78 μmol/mL) in the TG1 group. The TG2 group had reduced IL-6 levels by 26.81% (7.18 μmol/mL) and TG3 by 23.55% (7.50 μmol/mL). The administration of 250 mg/kg BW had the highest reduction effect, whereas the administration of diclofenac sodium reduced IL-6 levels by 24.10% (7.45 μmol/mL).

**Figure 3:**
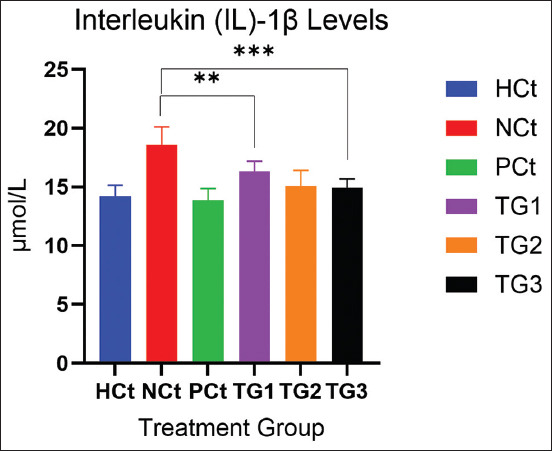
IL-1β levels after treatment. A significant increase (p < 0.05; *: indicates significance). Data are expressed as mean ± SD (n: 4). HCt=Healthy control, NCt=Negative control (CFA-induction only), PCt=Positive control (administration of sodium diclofenac 0.012 g/mL), TG1=Administration of 250 mg of *C. comatus* nanogel, TG2=Administration of 500 mg of *C. comatus* nanogel, TG3=Administration of 750 mg of *C. comatus* nanogel for 30 days. TG=Treatment groups, IL=Interleukin, CFA=Complete Freund’s Adjuvant, SD=Standard deviation, *C. comatus*=*Coprinus comatus*.

The NCt group had the highest IL-6 levels with 9.81 μmol/mL and the HCt group 7.05 μmol/mL. In addition to the decrease in TNF-α and IL-6, a decrease in IL-1β occurred in the group given *C. comatus* extract nanogels. The measurement results showed that *the C. comatus* nanogels were significantly (p < 0.05) able to reduce IL-1β levels after 30 days of administration. IL-1β levels are presented in Figure 3.

Figure 3 presents the decrease in IL-1β in all experimental groups. The highest decrease was observed in the PCt group (25.43% (13.87 μmol/mL), whereas the NCt group had the highest IL-1β levels, with 18.60 IL-1β. The experimental groups with the administration of *the C. comatus* nanogel exhibited reductions of 12.26% (TG1), 18.98% (TG2), and 20.50% (TG3), respectively.

## COX-2 measurement results

**Figure 4:**
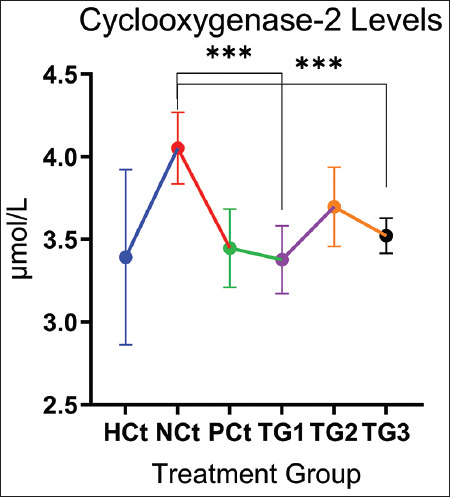
COX-2 levels after treatment. A significant increase (p < 0.05; *: indicates significance). Data are expressed as mean ± SD (n: 4). HCt=Healthy control, NCt=Negative control (CFA-induction only), PCt=Positive control (administration of sodium diclofenac 0.012 g/mL), TG1=Administration of 250 mg of *C. comatus* nanogel, TG2=Administration of 500 mg of *C. comatus* nanogel, TG3=Administration of 750 mg of *C. comatus* nanogel for 30 days. TG=Treatment groups, COX-2=Cyclooxygenase-2, CFA=Complete Freund’s Adjuvant, SD=Standard deviation, *C. comatus*=*Coprinus comatus*.

The COX-2 measurement was carried out to determine the anti-inflammatory effect of the *C. comtus* nanogels, apart from measuring the cytokine levels. The results of COX-2 measurements in the *C. comtus* nanogel group showed a significant reduction effect (p < 0.05). The COX-2 measurement results are presented in Figure 4.

The measurement results of COX-2 levels in the PCt group showed a decrease of 14.81% (3.45 μmol/mL) compared with the NCt group. The NCt group had the highest COX-2 levels with 4.05 μmol/mL. The decreases in COX-2 sequentially in groups administered with *C. comatus* nanogels from lowest to highest were TG2 8.64%, TG3 13.58%, and TG1 16.54%. The decrease in COX-2 concentration indicates that the inflammatory reaction in experimental animals decreases with the administration of *C. comatus* nanogel.

### Antibody measurement results (IgG and IgE)

**Figure 5:**
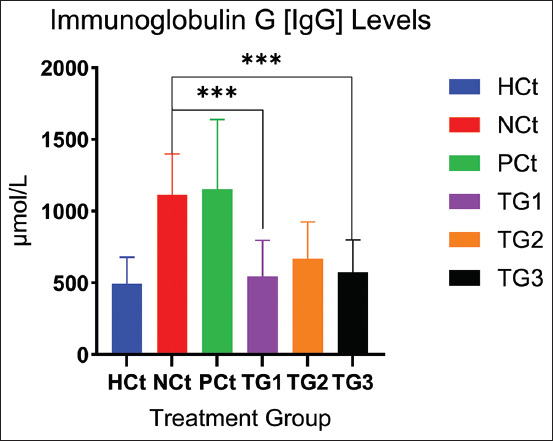
IgG levels after treatment. A significant increase (p < 0.05; *: indicates significance). Data are expressed as mean ± SD (n: 4). HCt=Healthy control, NCt=Negative control (CFA-induction only), PCt=Positive control (administration of sodium diclofenac 0.012 g/mL), TG1=Administration of 250 mg of *C. comatus* nanogel, TG2=Administration of 500 mg of *C. comatus* nanogel, TG3=Administration of 750 mg of *C. comatus* nanogel for 30 days. TG=Treatment groups, IgG=Immunoglobulin G, CFA=Complete Freund’s Adjuvant, SD=Standard deviation, *C. comatus*=*Coprinus comatus*.

Inflammatory reactions, which also involve immunological substances such as cytokines, also involve antibodies. In this study, we measured IgG and IgE antibodies. A significant decrease in antibodies, especially IgG (p < 0.05), occurred after administration of the *C. comatus* nanogel. IgG levels after administration of *the C. comatus* nanogel are presented in Figure 5.

Figure 5 shows the effect of *C. comatus* nanogel administration after 30 days of administration in all experimental groups. Nanogel administration decreased IgG levels, especially in the experimental groups, compared with the NCt group. The highest IgG decrease was observed in the TG1 group (51.04% [545.58 μmol/mL]), followed by the TG3 group (48.43% [574.73 μmol/mL]), whereas the TG2 group showed a decrease in IgG by 39.96% (669.02 μmol/mL). The highest IgG level was observed in the NCt group with 1114.44 μmol/mL, while the HCt group was 492.13 μmol/mL, the administration of nanogels at a dose of 250 mg/Kg BW had a decreasing effect that was close to the levels of the healthy/HCt group.

**Figure 6:**
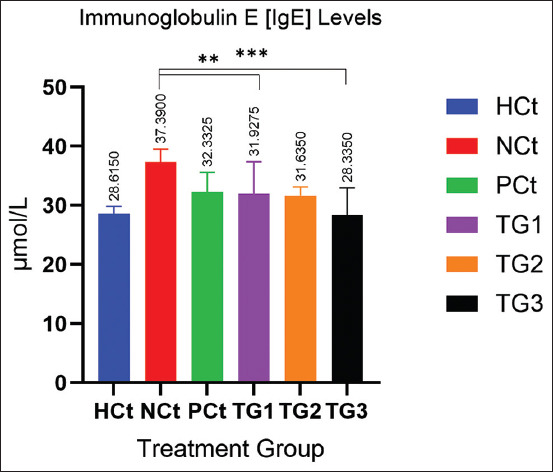
IgE levels after treatment. A significant increase (p < 0.05; *: indicates significance). Data are expressed as mean ± SD (n: 4). HCt=Healthy control, NCt=Negative control (CFA-induction only), PCt=Positive control (administration of sodium diclofenac 0.012 g/mL), TG1=Administration of 250 mg of *C. comatus* nanogel, TG2=Administration of 500 mg of *C. comatus* nanogel, TG3=Administration of 750 mg of *C. comatus* nanogel for 30 days. TG=Treatment groups, IgG=Immunoglobulin G, CFA=Complete Freund’s Adjuvant, SD=Standard deviation, *C. comatus*=*Coprinus comatus*.

In addition to the decrease in COX-2 and IgG levels, the administration of *C. comatus* ethanol extract nanogels significantly reduced IgE levels (p < 0.05) after 330 days of administration. The IgE measurement results are presented in Figure 6.

Figure 6 shows the effect of decreasing IgE levels after administration of *the C. comatus* extract nanogels. The highest IgE level was observed in the NCt group at 37.39 μmol/mL, whereas the IgE level of the healthy/HCt group was 28.62 μmol/mL. The decrease in IgE levels in the experimental groups given nanogels in order of the highest decrease effect was TG3 24.23% (28.33 μmol/mL), TG2 15.41% (31.63 μmol/mL), and TG1 14.61% (31.93 μmol/mL). In addition to the anti-inflammatory parameters used in this study consisting of pro-inflammatory cytokines and antibodies, the edema volume and AI of the CFA-induced feet of experimental animals measured for 30 days were also evaluated.

**Figure 7:**
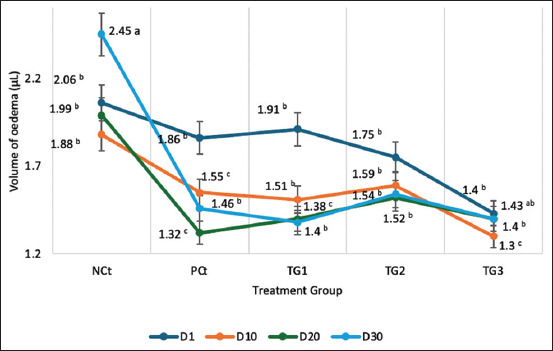
Paw edema volume of treatment group. Scores on each line marked with the same letters in the same column on the table are not significantly different at the levels (p < 0.05), data are expressed as mean ± SD (n: 4). HCt=Healthy control, NCt=Negative control (CFA-induction only), PCt=Positive control (administration of sodium diclofenac 0.012 g/mL), TG1=Administration of 250 mg/kg BW *C. comatus* nanogel, TG2=Administration of 500 mg/kg BW *C. comatus* nanogel, TG3=Administration of 750 mg/kg BW *C. comatus* nanogel for 30 days. TG=Treatment groups, CFA=Complete Freund’s Adjuvant, SD=Standard deviation, BW=Body weight, *C. comatus*=*Coprinus comatus*.

**Figure 8:**
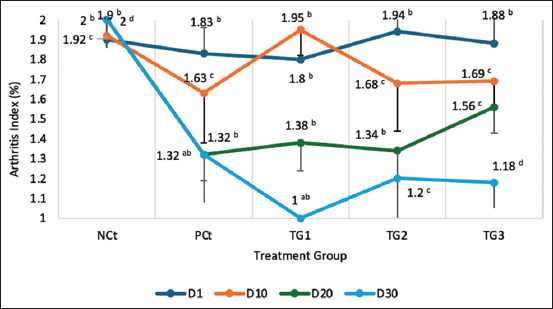
Arthritis index measurement results. Scores on each line marked with the same letters in the same column on the table were not significantly different at the levels (p < 0.05). Data are expressed as mean ± SD (n: 4). HCt=Healthy control, NCt=Negative control (CFA-induction only), PCt=Positive control (administration of sodium diclofenac 0.012 g/mL), TG1=Administration of 250 mg/kg BW *C. comatus* nanogel, TG2=Administration of 500 mg/kg BW *C. comatus* nanogel, TG3=Administration of 750 mg/kg BW *C. comatus* nanogel for 30 days. TG=Treatment groups, CFA=Complete Freund’s Adjuvant, SD=Standard deviation, BW=Body weight, *C. comatus*=*Coprinus comatus*.

The edema and AI measurements were evaluated every 5 days for 30 days. The results of edema volume measurements are presented in Figure 7, and the results of AI measurements are presented in Figure 8.

The results of the measurement of paw edema volume of experimental rats for 30 days after administration of *C. comatus* extract nanogels showed a significant decrease (Figure 7). The TG1 group showed the highest decrease with 42.42% (1.75–1 μL), followed by the TG3 group at 36.70% (1.88–1.19 μL) and the TG2 group at 30.94% (1.81–1.25 μL). The PCt group receiving diclofenac sodium showed a decreasing effect of 27.62% (1.81–1.31 μL), whereas in the NCt group, the volume of edema of the rat’s feet actually increased every week, with the highest increase of 3%.

### AI

Based on the results of measuring the AI during 30 days of measurement, it was found that the AI in the NCt group tended to increase, while in the group with *C. comatus* nanogel administration, it tended to decrease every week (Figure 8). This decrease in AI was significant (p < 0.05), especially in the nanogel group.

Figure 8. shows the results of AI measurements in all experimental groups for 30 days, showing that the NCt group tended to increase every week, with the highest increase being 15.92% (2.06–2.45 mL). The administration of diclofenac sodium in the PCt group showed a decrease of 21.51% (1.86–1.46 mL), whereas in the group given *C. comatus* nanogels in order from the smallest to the highest decreasing effect were TG3 2.15% (1.43–1.40 mL), TG2 12% (1.75–1.54 mL), and TG1 27.75% (1.91–1.38 mL).

## DISCUSSION

Animal models with CFA induction are widely utilized in research to assess and screen the efficacy of drugs or compounds derived from extracts for their anti-inflammatory and anti-arthritic properties [22]. Recent research by Ratnaningtyas *et al*. [11] indicates that *C. comatus* mushrooms exhibit significant anti-inflammatory and immunosuppressive effects in carrageenan-induced experimental animals by reducing pro-inflammatory cytokine levels, particularly IL-1β, within 14 days of extract administration.

CFA induction triggers chronic inflammation, leading to increased free radicals, elevated pro-inflammatory cytokine levels, and potential damage to inflammatory cells or tissues [23]. When evaluating the efficacy of herbal medicines or compounds with anti-inflammatory potential, it is crucial to consider more than just the reduction of pro-inflammatory cytokines, given the complex pathophysiology of RA. RA involves the continuous activation of intricate cellular processes that initiate an autoimmune response affecting the joints and potentially other organs.

The pathological mechanisms of RA are closely associated with T cells. Molecularly shared epitopes suggest that certain alleles with conserved sequences are implicated in RA pathogenesis. These alleles enable antigen-presenting cells to inaccurately present antigens to T cells, resulting in a T-cell-mediated autoimmune response that significantly contributes to the development and progression of RA [4].

The inflammation and proliferation of FLS can contribute to chronic inflammation, primarily driven by elevated PGE2 activity, which is stimulated by increased COX-2 enzyme activity. The induction of inflammatory agents, such as CFA, can lead to localized inflammation, resulting in greater edema volume and arthritis due to leukocyte infiltration at the inflammation site. In addition, the development of edema is closely associated with heightened levels of reactive oxygen species (ROS). This process is facilitated by the activation of protein kinase C (PKC).

During inflammatory conditions, enhanced vasodilation and increased permeability of the vascular membrane occur. Concurrently, the release of inflammatory mediators, including histamine from mast cells and serotonin, promotes PG synthesis, a process mediated by COX and lipooxygenase (LOX) enzymes. These biochemical events are initiated by PKC activation, which stimulates the phospholipase A2 and mitogen-activated protein kinase (MAPK) pathways, leading to the release of thrombosis activation factor (platelet-activating factor) and arachidonic acid [24].

The findings of this study indicate that the observed edema was attributable to increased PGE2 production, which resulted from elevated COX-2 enzyme activity. Moreover, the results demonstrated that the CFA-induced group exhibited significantly higher COX-2 levels, with the most pronounced increase observed in the NCt group.

Following CFA induction, pro-inflammatory cytokines such as TNF-α, IL-1β, and IL-6 exhibited a marked increase. Conversely, a reduction in these cytokine levels was observed in the group treated with *C. comatus* nanogels. Elevated IL-6 expression can further promote the production of other pro-inflammatory cytokines, including TNF-α and IL-1β. This escalation in cytokine activity stimulates polymorphonuclear cell (PMN) activity, contributing to the persistence of chronic inflammation. The heightened PMN activity also induces the expression of inducible nitric oxide synthase (iNOS), leading to an increased generation of reactive nitrogen species (RNS), such as NO^-^ and peroxynitrite anion (ONOO^-^) [1].

During inflammation, NO^-^ production is amplified due to elevated iNOS enzyme activity. Through the Fenton reaction, NO^-^ can react with other ROS like O_2_, resulting in the formation of more reactive free radicals. This interaction exacerbates inflammatory responses by promoting oxidative stress [25]. Shirani *et al*. [5] have also indicated that CFA induction not only elevates pro-inflammatory cytokines including TNF-α, INF-γ, IL-4, and IL-10 but also increases the production of PGE2. In RA, the increase in ROS levels during inflammation and arthritis is partly due to the release of myeloperoxidase from activated neutrophils [26].

The pathogenic mechanisms of RA are not solely linked to the innate and adaptive immune systems. Several pro-inflammatory cytokines activate specific signaling pathways related to RA, such as MAPK and nuclear factor kappa B (NF-κβ), which, in turn, stimulate the activation of mesenchymal cells and synoviocytes. Prominent cytokine mediators involved in this process include IL-1β, IL-6, and TNF-α. These mediators contribute to synovial inflammation, reduced lymphangiogenesis, and enhanced angiogenesis [6].

The administration of *C. comatus* nanogels demonstrated an ability to reduce COX-2 enzyme activity and subsequently inactivate the NF-κβ signaling pathway. Inactivation of NF-κβ hampers the formation and release of inflammatory mediators such as IL-1β and IL-6, resulting in diminished ROS and RNS production [1]. The triterpenoid compounds present in *C. comatus* nanogels contribute to this reduction in NF-κβ activity. These compounds exhibit anti-inflammatory properties by inhibiting cellular proliferation and suppressing NF-κβ signaling, thereby preventing the excessive release of inflammatory mediators like TNF-α [7]. Moreover, inhibiting NF-κβ concurrently decreases the synthesis of COX-2, iNOS, TNF-α, and IL-1β. This reduction in COX-2 and iNOS levels limits NO^-^ free radical formation, helping to mitigate the development of chronic inflammation [27].

The bioactive components of *C. comatus* also include rutin, a potent antioxidant that stabilizes free radicals and prevents their harmful effects [24]. In addition, polyphenol and flavonoid compounds within *C. comatus* can donate electrons (H^+^) to neutralize free radicals, thus averting oxidative stress and preventing lipid peroxidation of cell membranes, which can lead to organ damage [21]. Furthermore, *C. comatus* contains compounds from the flavonol group, including quercetin, rutin, and quercitrin; the chlorogenic acid group, such as quinic acid; and the flavone group, including apigenin, baicalein, and vitexin. These bioactive compounds function as exogenous antioxidants, disrupting lipid peroxidation processes under inflammatory conditions [28].

This study demonstrates that administering *C. comatus* nanogels significantly reduce pro-inflammatory cytokine levels, COX-2 enzyme activity, and serum antibody concentrations during the inflammatory response. In the early stages of RA, characterized by swelling and heightened antigen presence due to CFA induction, IgG activity is notably stimulated [29]. Previous research has established a positive correlation between bone damage, joint deformation in RA patients, and rheumatoid factors, including IgG autoantibodies. In animal models of RA, elevated IgG concentrations at joint sites have been observed. These IgG autoantibodies exacerbate joint conditions in experimental animals, contributing to increased inflammation [30].

IgE also plays a critical role at the onset of infection. IgE binds to its receptor, FcεRI, located on the surface of mast cells. FcεRI is the main receptor present on mast cells and basophils. This receptor functions to receive signals from IgE that binds to antigens coming from outside and triggers an inflammatory reaction. Increase in IgE levels can trigger enhanced histamine release by mast cells. Ratnaningtyas *et al*. [11] revealed that administering *C. comatus* ethanol extract to carrageenan-induced inflammatory model mice resulted in a 59.04% reduction in IgE levels at an extract dose of 500 mg/kg BW. The present study’s findings indicate decreased IgG and IgE levels following *C. comatus* nanogel administration suggests effective suppression of inflammation in experimental animals, thereby minimizing the progression toward chronic inflammation.

In contrast, the group not treated with *C. comatus* nanogels exhibited increased IgG and IgE levels, surpassing those observed in the HCt group. These results were further supported by a reduction in foot edema and a lower AI in all groups treated with *C. comatus* nanogels. In addition, prior research indicated that administering *Moringa rivae* extract to CFA-induced experimental animals reduced swelling by 78.56% at a dose of 600 mg/kg BW. This treatment also effectively decreased the levels of pro-inflammatory markers, including IL-10, IL-4, COX-2, TNF-α, IL-1β, and IL-6 [31].

This study revealed that the compounds present in *C. comatus* nanogels, including triterpenoids, polyphenols, tannins, flavonoids, alkaloids, phosphoric acid, and fatty acids, exhibit significant anti-arthritic and anti-inflammatory properties. These therapeutic effects are associated with a reduction in pro-inflammatory cytokines, inflammatory mediator enzymes, and antibodies. Previous studies on the use of mushrooms as herbal anti-inflammatory agents have shown similar outcomes, with *G. lucidum* nanogels demonstrating reductions in IgG, TNF-α, IL-1β, COX-2, and IL-6 levels. The *G. lucidum* nanogels were found to contain approximately 1.43 mg/g flavonoids, 2.66 g alkaloids, and 1.84 mg/g organic compounds [1].

In the present study, *C. comatus* nanogels were found to contain 1.51 mg/g flavonoids with antioxidant properties. In addition, other research has demonstrated that *C. comatus* possesses anti-inflammatory activity, attributed to its high polysaccharide content. The polysaccharides in *C. comatus* can effectively reduce TNF-α, COX-2, IL-6, and iNOS levels. The reduction in iNOS is particularly beneficial in minimizing the pathogenesis of RA, as it helps suppress the release of NO^-^ radicals. Moreover, *C. comatus* polysaccharides were observed to enhance enzymatic antioxidant levels, including superoxide dismutase, glutathione, and catalase, while decreasing malondialdehyde (MDA) levels, the final product of lipid peroxidation [32].

The anti-inflammatory effects of *C. comatus* hot water extract are also linked to its ability to reduce superoxide anion radicals. Compounds such as flavonoids, polyphenols, and α-tocopherol in *C. comatus* contribute to lowering O_2_^–^ radicals and reducing IL-6 levels. Ergothioneine (EGT), a compound found in *C. comatus*, was particularly effective in suppressing IL-6 release during inflammation. The study’s findings indicate that the inhibition of IL-6 was more pronounced with the addition of EGT compared to polysaccharides alone [33]. EGT also exhibits anti-diabetic, antioxidant, and anti-cancer activities [34].

The inhibition of NO^-^ production by *C. comatus* nanogels is critical in reducing the risk of RA pathogenesis related to inflammation. NO is known to promote oxidative stress and lipid peroxidation, which facilitate the progression of inflammation and increase the release of IL-6, IL-1β, and TNF-α [35]. Furthermore, triterpenoid compounds found in fungi possess anti-inflammatory activity, as evidenced by previous studies showing that triterpenoids in *G. lucidum* and *Ganoderma sinense* reduce IL-1β, TNF-α, and COX-2 levels and suppress the expression and activity of iNOS, COX-2, and NF-κB [36].

The bioactive compounds contained in *C. comatus* nanogels decrease the levels of pro-inflammatory cytokines and increase free radicals and autoantibodies that cause chronic inflammation [Figures 7 and 8]. This chronic inflammation causes edema of the paws of experimental mice and an increase in the AI [14]. *C. comatus* mushrooms contain polysaccharides, quercetin, rutin, ascorbic acid, polyphenols, and flavonoids with biological and pharmacological activities [37]. Polysaccharide compounds from fungi are known to play antiarthritic activities, especially β-glucans. The β-glucan compound comprises two leading structural chains, β-1,3 and β-1,4 glucopyranosyl. These two main structures play anti-inflammatory activities [38]. β-1.6 D-glucan compounds also have anti-arthritic activities [39]. With the increase in ROS that occurs in chronic inflammation in RA patients, β-glucan acts as an antioxidant by suppressing superoxide anion radicals (O_2_^-^), hydroxyl radicals (OH^-^), and peroxyl radicals (NOO^-^) [12]. Suppressed free radicals can minimize the risk of oxidative stress and stimulate the release of inflammatory mediators such as TNF-α and IL-1. The results of this study also show the flavonoid content in *C. comatus* nanogels, where flavonoids act as exogenous antioxidants that can suppress ROS to prevent lipid peroxidation reactions and damage to tissues or organs. Previous studies have also shown that the induction of inflammatory agents, such as carrageenan, triggers inflammation that causes edema. Several released inflammatory mediators such as PG, COX, and NO contribute to the severity of RA [22]. Jayasuriya *et al*. [40] have also shown that administering *Pleurotus ostreatus* extracts with β-glucan content can reduce the production of PG produced by tissue macrophages and the formation of NO and free radicals that can cause inflammation severity. The anti-inflammatory effect of *C. comatus* bioactive compounds such as triterpenoids is to reduce IgG levels. IgG has an autoantibody mechanism, in the pathogenesis of RA, autoantibodies increase, and IgG deposition in the joints increases. This was shown in *in vivo* experiments on RA model animals where, in addition, there was a decrease in levels of TNF-α, IL-6, IL-10, IL-6, IL-8, and IL-1β [30]. *C. comatus* also contains flavonoids which have a role as anti-inflammatory, apart from being an antioxidant. Flavonoids as anti-inflammatory agents occur through the mechanism of inhibition of the enzymes LOX, iNOS, and COX-2, and they inhibit NF-B activity. Inhibition of iNOS has a decreasing effect on NO^-^ levels, thus preventing lipid peroxidation reactions and minimizing cell and tissue damage. Polyphenol compounds are also present in *C. comatus* nanogels. Polyphenols, apart from being H^+^ donors to free radicals, also act as anti-inflammatory agents in RA conditions. Polyphenols inhibit the transcription factor NF-kB, activating protein-1, and minimize MAPK activity [41]. Previous research also showed that *G. lucidum* nanogels containing flavonoids, triterpenoids, polyphenols, and carboxylic acid could exert anti-inflammatory effects in RA animal models. The effect is to reduce the levels of COX-2, IL-1β, TNF-α, and IL-6. Flavonoid inhibition of COX-2 causes NF-kB activity to become inactive, resulting in a decrease in the production and release of inflammatory mediators such as TNF-α and IL-6. The results also showed that triterpenoid, polyphenol, and flavonoid compounds had a positive effect on reducing COX-2 and IL-1β levels. Inhibition of NO^-^ activity also occurs because triterpenoid compounds inhibit iNOS activity; a decrease in NO^-^ can prevent peroxidation activity that can produce other reactive radicals such as ONOO^-^ and the Fenton reaction. With a decrease in free radicals, inhibition of the activity of inflammatory mediator enzymes such as COX-2 and LOX can prevent the pathogenesis of RA due to inflammatory reactions [1].

Based on the study’s findings, the administration of the three different doses of nanogels resulted in varying effects on the reduction of pro-inflammatory cytokines, inflammatory enzyme mediators, and antibody levels as well as on arthritis and edema indices. The study’s results do not conclusively demonstrate that the highest nanogel dose always produces the greatest effect. For instance, in the case of TNF-α, IL-6, COX-2, IgG, and AI values, the 250 mg/kg BW dose exhibited a slightly higher effect compared to the 500 mg/kg BW dose. Conversely, the 750 mg/kg BW dose showed the most significant reduction in IL-1β, IgE, and edema levels.

The differing effects of nanogels across groups with varying doses are influenced not only by factors such as the distribution of bioactive compounds within the preparation, the quality of the preparation, and technical aspects during the extraction process but also by the biological and biochemical characteristics of the experimental animals. Studies involving different doses often reveal varying effects across different parameters. For example, in an anti-diabetic evaluation of the ethyl acetate extract of *C. comatus*, a 250 mg dose demonstrated the highest efficacy in reducing blood glucose levels and enhancing insulin levels when compared to a 500 mg dose [42]. In addition, the 500 mg *C. comatus* ethanol extract exhibited the best effect in lowering insulin levels relative to the 250 mg dose [10]. In an anti-inflammatory study, the 750 mg *C. comatus* nanogel achieved the greatest IgG reduction effect compared to the 250 mg and 500 mg doses [1].

Other contributing factors to the observed differences in the reduction and elevation of measured parameters, apart from the quality of the preparation, include the types of preparations used. This is evident as studies utilizing extracts and nanogels tend to produce distinct effects.

The potential of nanogels derived from *C. comatus* mushrooms has demonstrated effectiveness as anti-arthritis and anti-inflammatory agents by reducing pro-inflammatory cytokine levels and COX-2 enzyme activity, suppressing antibody levels in serum, and decreasing edema and arthritis in the feet of experimental animals. Various identified compounds within *C. comatus* mushrooms present opportunities for further exploration by testing isolated single compounds for their potential therapeutic benefits.

The development of herbal medicines with minimal side effects is crucial due to their favorable therapeutic effects, high efficiency, and low toxicity. To ensure that alternative herbal medicines can be effectively and efficiently utilized by individuals with RA [14], future research on *C. comatus* nanogel could focus on developing microencapsulant formulations that are easier to apply. In addition, incorporating free radical parameters, such as NO^-^, superoxide (O_2_^-^), MDA levels, and histological parameters, would provide a more detailed understanding of its therapeutic effects.

## CONCLUSION

This study evaluated and investigated the therapeutic potential of *C. comatus* nanogels as anti-arthritic and anti-inflammatory agents with various anti-arthritic and anti-inflammatory parameters in CFA-induced inflammatory model rats. The findings of this study revealed that *C. comatus* nanogels were able to reduce the levels of pro-inflammatory cytokines, inflammatory mediator enzymes, and antibodies that play a role in inflammation, as well as the AI and edema volume. RA treatment that focuses on reducing the effects of pro-inflammatory cytokines and inflammatory enzyme mediators is important so that the pathogenicity of RA disease does not progress due to ongoing inflammatory reactions. In addition, the antibody parameters measured can also prove that *C. comatus* has good activity in minimizing the risk of inflammation in RA, considering that IgE and IgG antibodies act as triggers for inflammatory reactions and the release of inflammatory mediators. The use of mushrooms as herbal medicine candidates for RA is also a good discovery, considering that there are not many herbal medicine products from mushrooms. The contents of *C. comatus* such as triterpenoids, flavonoids, alkaloids, saponins, and mineral acids provide a good therapeutic effect in reducing TNF-α, IL-6, and IL-1β, COX-2, and antibodies (IgG and IgE) and reducing edema volume and the AI.

However, this study has limitations, such as testing that is still limited to *in vivo* experiments with experimental animals and has not been applied to humans. The identification of *C. comatus* compounds also requires high-resolution mass spectrometry to identify a more complete metabolomic profile. To determine the anti-arthritic and anti-inflammatory effects, it can also be done again with histopathological evaluation to know the pathogenesis of RA and the effect of preventing damage due to RA and inflammation from *C. comatus* nanogels. In addition, it is necessary to test the safety of nanogel products through long-term toxicity testing.

Further research can be conducted by developing preparations in other forms with more specific compound identification to obtain data on the effectiveness of *C. comatus* as a therapy with more diverse activities. In addition, exploration and testing in humans are needed to determine its effectiveness as an RA herbal medicine candidate. Acute and chronic toxicity testing also needs to be performed to obtain information on the potential of safe therapies for the treatment of RA.

This study provides valuable insight into the development of herbal medicines from mushrooms for the treatment of RA, with the main effect being the suppression of pro-inflammatory cytokines and inflammatory mediator enzymes. The use of mushrooms as natural resources that are safer, have fewer side effects, and are easily available can also be further developed for RA treatment.

## AUTHORS’ CONTRIBUTIONS

NIH and FH: For writing and improving the manuscript, conducted the research design, laboratory experiments, and sample analysis. NIH: Analyzed the ELISA samples, and FH: Analyzed the bioactive compound samples and the statistical analysis. All authors have read and approved the final manuscript.
